# miR‐542‐3p prevents ovariectomy‐induced osteoporosis in rats via targeting SFRP1

**DOI:** 10.1002/jcp.26430

**Published:** 2018-04-16

**Authors:** Xiguang Zhang, Yun Zhu, Chuanlin Zhang, Jianping Liu, Tianming Sun, Dan Li, Qiang Na, Cory J. Xian, Liping Wang, Zhaowei Teng

**Affiliations:** ^1^ Department of Orthopedic Surgery The People's Hospital of Yuxi City The 6th Affiliated Hospital of Kunming Medical University Yuxi Yunan China; ^2^ Health Screening Center, The People's Hospital of Yuxi City The 6th Affiliated Hospital of Kunming Medical University Yuxi Yunan China; ^3^ Department of Nuclear Medicine, The People's Hospital of Yuxi City The 6th Affiliated Hospital of Kunming Medical University Yuxi Yunan China; ^4^ Department of Clinic Laboratory, The People's Hospital of Yuxi City The 6th Affiliated Hospital of Kunming Medical University Yuxi Yunan China; ^5^ Sansom Institute for Health Research, School of Pharmacy and Medical Sciences University of South Australia Adelaide SA 5001 Australia

**Keywords:** differentiation, miRNA, osteoporosis, SFRP1

## Abstract

Secreted frizzled‐related protein‐1 (SFRP1) is a negative regulatory molecule of the WNT signaling pathway and serves as a therapeutic target for bone formation in osteoporosis. In this study, we first established an ovariectomized (OVX) rat model to simulate postmenopausal osteoporosis and found significant changes in miR‐542‐3p and sFRP1 expression by RNA sequencing and qRT‐PCR. In addition, there was a significant negative correlation between miR‐542‐3p and sFRP1 mRNA levels in postmenopausal women with osteoporosis. We found that miR‐542‐3p inhibited the expression of sFRP1 mRNA by luciferase reporter assay. When the miR‐542‐3p binding site in sFRP1 3'UTR was deleted, it did not affect its expression. Western blot results showed that miR‐542‐3p inhibited the expression of SFRP1 protein. The expression of SFRP1 was significantly increased in osteoblast‐induced mesenchymal stem cells (MSC), whereas the expression of miR‐542‐3p was significantly decreased. And miR‐542‐3p transfected MSCs showed a significant increase in osteoblast‐specific marker expression, indicating that miR‐542‐3p is necessary for MSC differentiation. Inhibition of miR‐542‐3p reduced bone formation, confirmed miR‐542‐3p play a role in bone formation in vivo. In general, these data suggest that miR‐542‐3p play an important role in bone formation via inhibiting SFRP1 expression and inducing osteoblast differentiation.

## Graphical abstract

sFRP1 and miR‐542‐3p were negatively correlated in postmenopausal osteoporosis patients. sFRP1 was a target of miR‐542‐3p. miR‐542‐3p play an important role in bone formation by targeting SFRP1 and inducing osteoblasts differentiation.

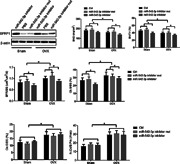

## 1 INTRODUCTION

Osteoporosis is a bone metabolic disease characterized by a decrease in bone mass and bone mineral density (BMD), and a degradation of bone tissue microstructures, which lead to an increase in bone fragility and fracture incidence (Kanis et al., 2013; Roux & Briot, 2017). The main pathogenesis of osteoporosis is caused by bone remodeling disorders, which are due to osteoclast‐mediated bone resorption rates higher than osteoblast‐mediated bone formation (Bidwell, Alvarez, & Childress, 2013). The incidence of osteoporosis in the elder population is higher (senile osteoporosis) (Li et al., 2017). In particular, postmenopausal women (postmenopausal osteoporosis) are prone to osteoporosis because of estrogen deficiency is associated with excessive bone resorption and poor bone formation (Epstein, 2006; Heiss et al., 2012). However, the detailed mechanisms of estrogen deficiency in postmenopausal osteoporosis have not been fully understood.

The canonical Wnt/β‐catenin signaling pathway plays a vital role in activating bone formation and resorption genes transcription, and then regulate osteoblast differentiation, proliferation, survival, and bone formation (Zhang & Drake, 2012). SFRP1 is homologous to the extracellular cysteine‐rich domain of the WNT receptor Frizzled, but lacks the intracellular and transmembrane domains (Yang et al., 2009). It has been reported that competition with extracellular Wnt binds to the Frizzled receptors, and further antagonizes Wnt signaling by direct binding to Wnt proteins (Gaur et al., 2006; Hausler et al., 2004). Previous studies have shown that SFRP1 plays an important role in osteoblast differentiation, trabecular bone formation and bone fracture healing (Bodine et al., 2004; Bodine, Seestaller‐Wehr, Kharode, Bex, & Komm, 2007; Gaur et al., 2006, 2009). Thus, the development of inhibitors against SFRP1 is a viable method of stimulating bone formation in metabolic bone diseases, osteoporosis and aging (Baron & Rawadi, 2007; Gaur et al., 2009).

MicroRNAs (miRNAs) is a class of small non‐coding (18–25 nucleotides) single‐stranded RNAs that binds to an incomplete or complete base pairing of a specific sequence in the 3′ untranslated regions (3′UTRs) or CDS of mRNAs, then induces either translational repression or cleavage of the target mRNAs (Noma et al., 2004; Schramke & Allshire, 2003). MiRNAs play important roles in a variety of biological processes, such as cell proliferation, differentiation, apoptosis, and tumorigenesis (Yang et al., 2013; Yin et al., 2013; Zhang, Wei, & Xu, 2013). Recently, numerous studies have revealed that miRNAs play critical roles in bone homeostasis. For example, miR‐433‐3p plays an critical role in DKK1/WNT/beta‐catenin pathway by reducing DKK1 expression and inducing osteoblast differentiation (Tang, Lin, Wang, & Lu, 2017). MiR‐23a cluster regulates osteoblast differentiation by attenuating *Prdm16* expression level to modulate the TGF‐β signaling pathway (Zeng et al., 2017). However, the role of miRNAs in the pathogenesis of osteoporosis remains to be further studied.

In this study, we found that sFRP1 and miR‐542‐3p were negatively correlated in postmenopausal osteoporosis patients. Then, miRNA target analysis and experiments showed that sFRP1 was a target of miR‐542‐3p. Further studies have shown that miR‐542‐3p plays a vital role during the process of MSC differentiation. In general, our study show that miR‐542‐3p play an important role in bone formation by targeting SFRP1 and inducing osteoblasts differentiation.

## 2 RESULTS

### 2.1 MiR‐542‐3p is differentially expressed and negatively correlated with SFRP1 in postmenopausal osteoporotic patients

**Figure 1:**
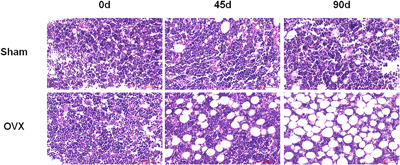
OVX surgery leads to dramatically decreased bone loss in rats. Histological sections of femur in rats of the sham and OVX groups. Sham: sham control group; OVX: the ovariectomized rats group; Scale bar, 100 μm. All sections were stained with HE

We chose estrogen deficient ovariectomized (OVX) rats model to mimic postmenopausal osteoporosis, because it is close to postmenopausal bone loss (Kalu, 1991). A total of 45 or 90 days after surgery, the bone mineral density (BMD) of right femoral in OVX rats was lower than that in sham operation group (Sham) (*p *< 0.05) (Table 1). Compared with sham rats, OVX rats also showed increased trabecular separation (Figure 1). It indicated that ovariectomy‐induced osteoporosis in rat model has been established.

**Table 1:** Bone mineral density of the femur

	0 d (mg/cm^2^)	45 d (mg/cm^2^)	90 d (mg/cm^2^)
Sham	182.44 ± 19.24	175.17 ± 15.15	168.06 ± 17.67
OVX	186.88 ± 20.45	140.70 ± 18.93	126.32 ± 14.90
*p* value	>0.05	<0.05	<0.05

**Figure 2:**
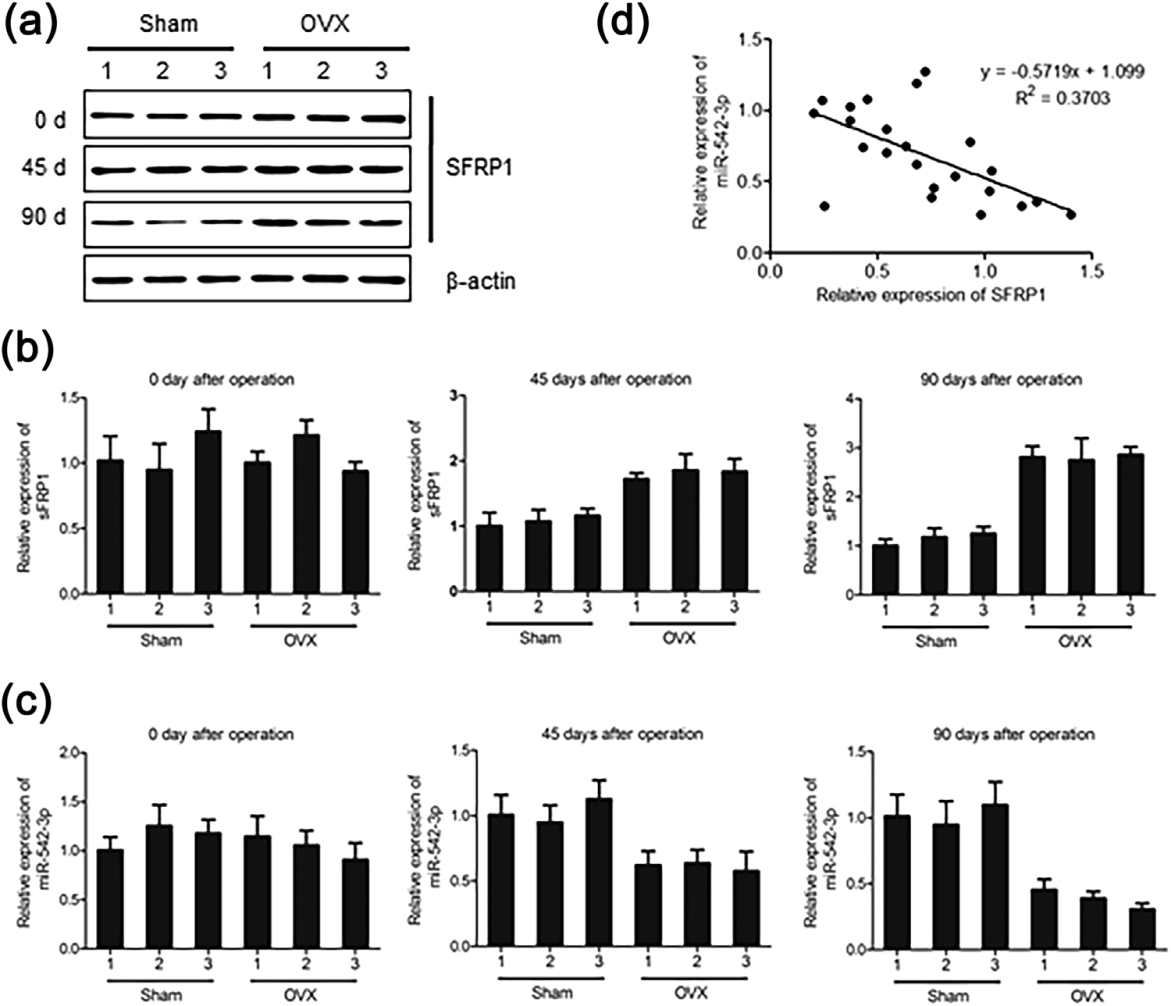
Expression of SFRP1 and miR‐542‐3p is significantly regulated in ovariectomized rats. (a–c) Expressions of SFRP1 protein (a), sFRP1 mRNA (b), and miR‐542‐3p (c) in MSCs from OVX rats. *p < 0.05 compared with Sham rats. (D) Correlation analysis of miR‐542‐3p and sFRP1 expressions in postmenopausal women with osteoporosis. SFRP1, secreted frizzled‐related protein 1. We have drawn the correlation formula: *y* = −0.5719x + 1.099 (x: expression level of sFRP1, *y*: expression level of miR‐542‐3p) (*n* = 23). All data were presented as mean ± S.E.M. from three independent experiments. *p < 0.05

To compare the mRNAs expression profiles in the MSCs from OVX rats and Sham rats, three subjects of each subgroup were selected for RNA‐seq assays. mRNAs with log_2_(fold change) >3‐fold were determined as putative candidates. As shown in Table 2, six mRNAs were up‐regulated and 22 mRNAs were down‐regulated. In the dramatically up‐regulated mRNAs, SFRP1 is an antagonist of Wnt signaling and play an important role in maintaining bone homeostasis (Baron & Rawadi, 2007; Gaur et al., 2009). So we selected sFRP1 as a candidate gene. The expression of SFRP1 was detected in three subjects using western blot and qRT‐PCR assay, and the results were consistent with RNA‐seq expression (Figures 2a and b). Meanwhile, we compared the expression of microRNAs in the MSCs from OVX rats and Sham rats (Supplementary Table SI). Among microRNAs that contain the binding sites of sFRP1 (Supplementary Table SII), the expression of miR‐542‐3p is significantly downregulated in the OVX group (Figure 2c). Finally, the relationship between miR‐542‐3p and sFRP1 expression levels in postmenopausal osteoporotic patients was analyzed and plotted on the graph shown in Figure 2d. Correlation analysis showed a significant negative correlation between sFRP1 and miR‐542‐3p expression (Figure 2d).

**Table 2:** Significantly regulated mRNA in the MSCs from ovariectomized rats

Gene name	Log2 (Fold change)	*q* value
Areg	6.52423	1.09E‐03
Bmpr1b	4.51201	6.26E‐05
Gdf10	4.34626	7.46E‐08
Gpnmb	3.79445	2.19E‐04
Sfrp1	3.56823	4.30E‐05
Bmp6	3.29682	3.47E‐03
Npnt	−3.11794	8.66E‐03
Vcan	−3.29220	1.80E‐05
Fbn2	−3.52257	6.77E‐04
Satb2	−3.68697	3.70E‐05
Id4	−3.76725	1.07E‐05
Spp1	−3.82948	1.99E‐06
Sfrp2	−3.94169	1.89E‐03
Wnt10b	−4.00330	6.94E‐05
Npnt	−4.07834	2.95E‐03
Vcan	−4.08130	7.80E‐03
Rassf2	−4.41967	5.40E‐07
Nog	−4.55849	3.19E‐04
Penk	−4.60582	3.09E‐06
Wnt4	−4.94307	1.52E‐03
Gdpd2	−5.13725	6.75E‐08
Alpl	−5.38651	1.80E‐10
Sp7	−5.53967	6.20E‐08
Pth1r	−5.63211	6.05E‐12
Penk	−5.67360	1.58E‐09
Dlx5	−6.81663	2.50E‐11
Bmp8a	−7.33662	1.54E‐08
Sp7	−7.61160	1.75E‐12

### 2.2 MiR‐542‐3p directly targets sFRP1

It has been demonstrated that miRNAs regulate the mRNA expression of by binding to the 3′‐UTR or amino acid coding sequence (CDS) of target gene (Tay, Zhang, Thomson, Lim, & Rigoutsos, 2008). Based on the bioinformatics online analysis software (TargetScan), miR‐542‐3p was predicted to target in the 3′UTR of sFRP1.

**Figure 3:**
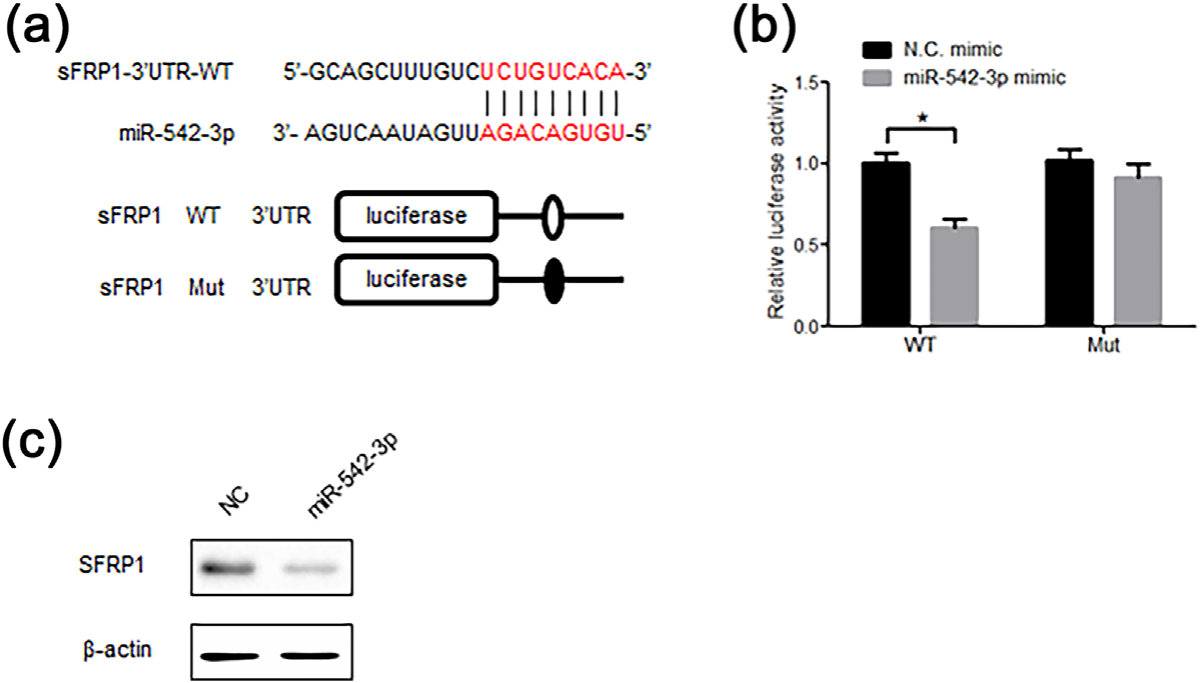
MiR‐542‐3p directly targets sFRP1. (a) Schematic diagram of miR‐542‐3p target site in the 3′ UTR of sFRP1 mRNA are shown in complementary pairing. (b) The luciferase reporter carrying pGL3‐sFRP1‐3′UTR‐WT or pGL3‐sFRP1‐3′UTR‐Mut was co‐transfected with the miR‐542‐3p mimic or the control in HEK293T cells for 48 hr, and luciferase activity assay was performed. Firefly luciferase values normalized for Renilla luciferase were presented. (c) HEK293T cells were transfected with miR‐542‐3p mimic, or the respective control for 48 hr. Western blot was used to detect the protein level of SFRP1. The data were from three independent experiments (*n* = 3). Data are shown as means ± SD. *p < 0.05 versus control

To investigate whether miR‐542‐3p is directly targeted to SFRP1, we firstly constructed luciferase reporter vectors which contained the predicted miRNA binding site of sFRP1 3'UTR or deleted mutant (Figure 3a). Then we introduced the luciferase expression vector containing the 3′UTR of sFRP1 (pGL3‐sFRP1‐3′UTR‐WT) and miR‐542‐3p into HEK293T cells, and measured the level of luciferase enzyme activity, which reflected the effects of miR‐542‐3p on luciferase translation. The results showed that overexpression of miR‐542‐3p suppressed the luciferase activity of the sFRP1 3′UTR reporter gene (Figure 3b). The deletion of the 9 nt continuous site complementary to miR‐542‐3p in the 3′UTR of sFRP1 (pGL3‐sFRP1‐3′UTR‐Mut) eliminates this inhibition, which confirmed the specificity of this action (Figure 3b).

In order to identify the effect of miR‐542‐3p on sFRP1 expression, miR‐542‐3p was transfected into HEK293T cells and the protein level of SFRP1 was measured by western blot. Overexpression of miR‐542‐3p significantly inhibited the protein level of endogenous SFRP1 compared to the control (Figure 3c).

These results indicate that sFRP1 is the target gene for miR‐542‐3p.

### 2.3 MiR‐542‐3p increased osteogenic differentiation

**Figure 4:**
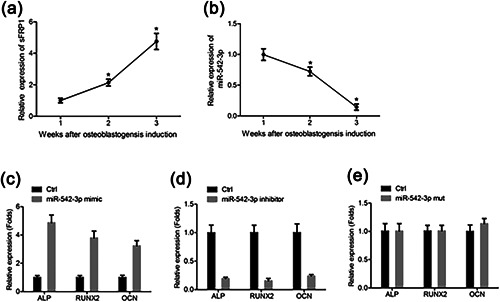
MiR‐542‐3p is essential in MSC differentiation. (a) Expression of sFRP1 during osteoblast induction from rats MSCs (*n* = 3). Data were normalized to β‐actin mRNA at each time point. Expression of sFRP1 at 1 week was normalized to 1. (b) Expression of miR‐542‐3p during osteoblast induction from rats MSCs (*n* = 3). Data were normalized to U6 RNA at each time point. Expression of miR‐542‐3p at 1 week was normalized to 1. (c–e) MSCs cells were transfected with miR‐542‐3p mimic (c), miR‐542‐3p inhibitor (d), miR‐542‐3p mutant (e), or their separate negative control. Expression of specific markers for osteoblasts (ALP, RUNX2 and OCN) was measured by qRT‐PCR (*n* = 3). β‐actin was used as the internal control. The data were from three independent experiments (*n* = 3). *p < 0.05

We first isolated the MSC from the rats and then induced to differentiate into osteoblasts. The expression of miR‐542‐3p and sFRP1 was measured from 1 to 3 weeks after induction. Osteoblast‐induced cells showed a significant increase in the expression level of sFRP1 from 1 to 3 weeks (Figure 4a). In contrast, the expression level of miR‐542‐3p was significantly reduced from 1 to 3 weeks, and the expression level of miR‐542‐3p was the lowest at 3 weeks after induction (Figure 4b).

Then, we detected the expression of specific markers for osteoblasts (ALP, RUNX2, and OCN) by qRT‐PCR in MSCs transfected with control miRNA, miR‐542‐3p, miR‐542‐3p inhibitor, or miR‐542‐3p mutant. Compared with the control group, miR‐542‐3p significantly increased the expression of osteoblast specific markers (*p* < 0.05) (Figure 4c), whereas miR‐542‐3p inhibitor allowed these osteoblast specific markers sharply decreased (*p* < 0.05) (Figure 4d). Moreover, this promotion was eliminated in miR‐542‐3p mutant transfected MSCs (Figure 4e).

### 2.4 MiR‐542‐3p promotes bone formation in vivo

**Figure 5:**
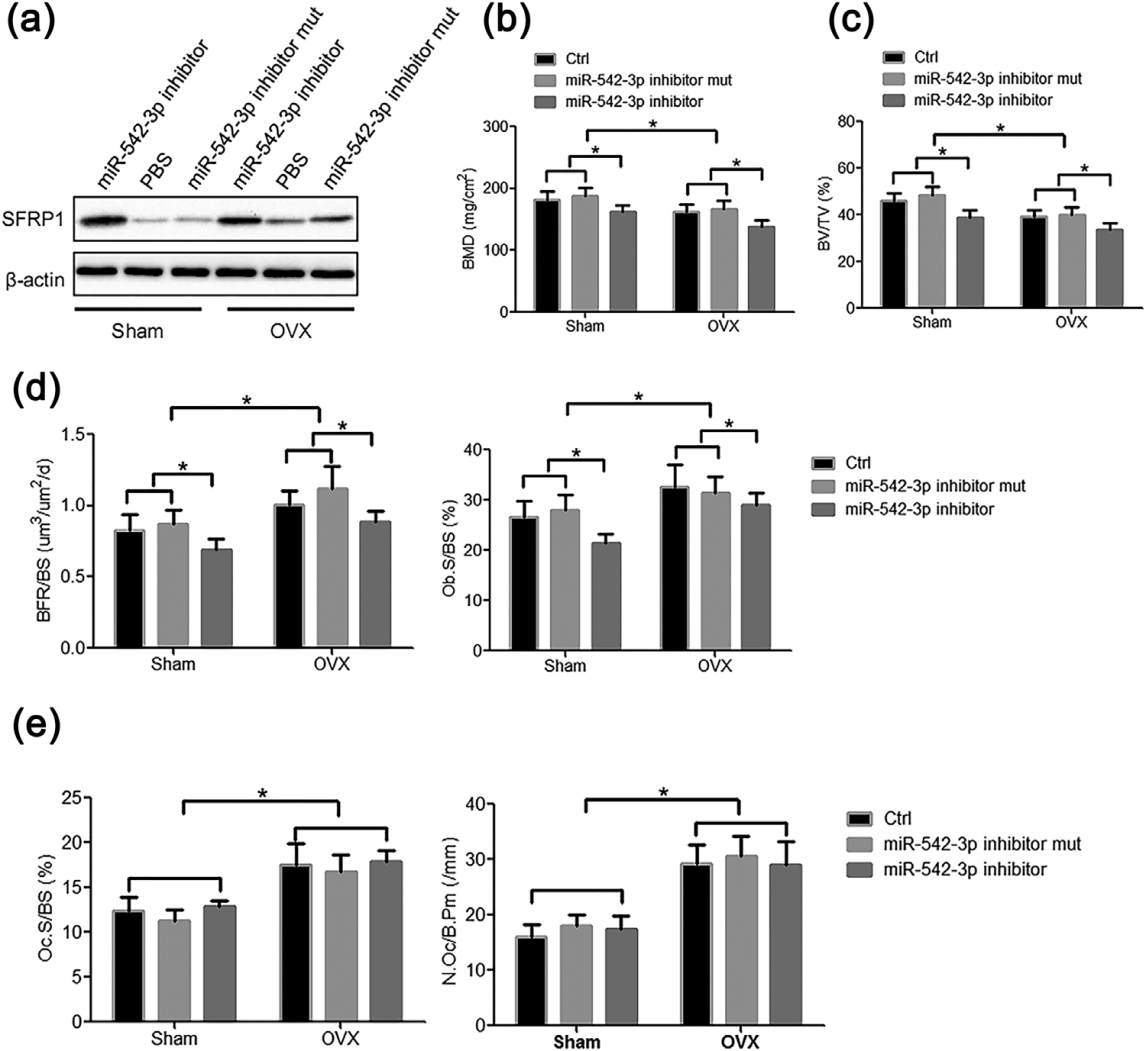
MiR‐542‐3p inhibitor‐treated rats showed decreased bone mass and reduced osteoblast activity. (a–e) Wild‐type rats were ovariectomized or sham surgery. After 4 weeks, rats were injected with PBS, miR‐542‐3p inhibitor, or miR‐542‐3p inhibitor mutant, and bones were harvested at 6 weeks after the first injection. (a) Expression of SFRP1 protein was analyzed by western blot. (b) Bone mineral density (BMD) of femurs was measured. (c) Structure parameter bone volume per tissue volume ratio (BV/TV) was measured by micro‐CT. (d) Bone formation rate over bone surface area (BFR/BS) and osteoblast surface area over bone surface area (Ob.S/BS) were measured by histomorphometric analysis. (E) Osteoclast surface area over bone surface area (Oc.S/BS) and the number of osteoclasts per bone perimeter (N.Oc/B.Pm) were measured by histomorphometric analysis. *p < 0.05. Table 1 Bone mineral density of the femur

In order to investigate the function of miR‐542‐3p in vivo, we used ovariectomy (OVX) rats to simulate postmenopausal status. Rats undergoing Sham surgery or OVX were given miR‐542‐3p inhibitor by single tail vein injection. Phosphate buffer solution (PBS) or miR‐542‐3p inhibitor mutant (mut) was used as a control. By Western blot, miR‐542‐3p inhibitor‐treated rats showed a higher expression level of SFRP1 protein compared with PBS or miR‐542‐3p inhibitor mutant‐treated Sham rats or OVX rats (Figure 5a).

Then a significant decrease in BMD was also detected. The results showed that miR‐542‐3p inhibitor treated rats exhibited a significant decrease in femur BMD compared to rats treated with miR‐542‐3p inhibitor mutant or PBS. OVX rats treated with miR‐542‐3p inhibitor, miR‐542‐3p inhibitor mutant or PBS also presented reduced femur BMD, whereas miR‐542‐3p inhibitor treated rats showed minimal BMD (Figure 5b).

Micro‐CT was used to quantify the bone volume/tissue volume ratio (BV/TV). This assay revealed that in Sham rats, a significant decrease in BV/TV was detected in miR‐542‐3p inhibitor‐treated rats compared to rats treated with PBS or miR‐542‐3p inhibitor mutant (*p* < 0.05) (Figure 5c). OVX rats treated with PBS or miR‐542‐3p inhibitor mutant also exhibited a significant decrease in BV/TV, while the lowest in the miR‐542‐3p inhibitor treated OVX rats (Figure 5c).

Compared with healthy controls, the femur bone mineral density of OVX rats significantly decreased. Next, we measured several parameters of femurs bone formation by histological analysis, including the bone formation rate on each bone surface (BFR/BS) and the osteoblast surface on each bone surface (Ob.S/BS). For BFR/BS and Ob.S/BS, statistically significant reductions were detected between the PBS or miR‐542‐3p inhibitor mutant and miR‐542‐3p inhibitor‐treated OVX rats or Sham rats (*p* < 0.05) (Figure 5d). Furthermore, a statistically significant increase in BFR/BS and Ob.S/BS was detected in OVX rats, compared to the sham group (*p* < 0.05) (Figure 5d). The bone resorption parameters of rat femur were also measured by histological analysis, including the osteoclast surface on each bone surface (Oc.S/BS) and the number of osteoclasts per bone perimeter (N.Oc/B.Pm). There was no statistically significant difference between PBS or miR‐542‐3p inhibitor mutant and miR‐542‐3p inhibitor‐treated OVX rats or control rats, indicating that osteoclast bone resorption was not affected (Figure 5e). However, OVX rats showed a statistically significant increase in Oc.S/BS and N.Oc/B.Pm compared to control rats in all constructs (*p* < 0.05).

## 3 DISCUSSION

Osteoporosis is a metabolic disorder characterized by osteoblast formation and osteoclast resorption imbalance, leading to skeletal fragility and fracture susceptibility. Ovarian hormone deficiency is a major risk factor for postmenopausal women with osteoporosis. It is well known that the OVX rats are the best animal model for the research of postmenopausal osteoporosis in women (Hartke, 1999). In the present study, we indicate that miR‐542‐3p is a novel miRNA that could ameliorate ovariectomy‐induced osteoporosis in rats by directly targeting sFRP1.

There is a growing evidence that miRNAs play key roles in both normal biological processes and the pathogenesis of human diseases through post‐transcriptional regulation of gene expression (Lamouille, Subramanyam, Blelloch, & Derynck, 2013). Several decades of miRNAs are considered necessary for bone formation (Lian et al., 2012). In this study, we found a higher level of sFRP1 in MSCs from OVX rats and a lower level of miR‐542‐3p in serum of OVX rats compared with Sham rats. Additionally, a correlation analysis further confirmed that miR‐542‐3p and sFRP1 were negatively correlated in postmenopausal osteoporotic patients. These results indicated that miR‐542‐3p may play a vital role in osteogenic differentiation of MSCs.

Previous studies have shown that miR‐542‐3p is a tumor suppressor that is abnormally down‐regulated in several human cancers. MiR‐542‐3p reduced cell viability, proliferation, tumor angiogenesis, invasion and metastasis, and induced apoptosis by targeting mRNAs, such as Survivin, FZD7, PIM1, cortactin, angiopoietin‐2, etc (Althoff et al., 2015; He et al., 2014; Long et al., 2016; Rang, Yang, Wang, & Cui, 2016; Wu et al., 2017). However, miR‐542‐3p may function as an oncogene by targeting VANGL2 during osteosarcoma pathogenesis (Li, Liu, Pei, Wang, & Lv, 2015). Recently, Farre et al reported that elevated miR‐542‐3p/5p may lead to muscle atrophy in ICU patients through promoting mitochondrial dysfunction and activation of SMAD2/3 phosphorylation (Farre Garros et al., 2017). However, the role of miR‐542‐3p in osteoporosis is unknown.

Here, we found that miR‐542‐3p regulates the osteogenesis differentiation of rat MSCs by directly targeting sFRP1. SFRP1 is an antagonist of Wnt signaling, which is an important pathway to maintain bone homeostasis. It has been reported that overexpression of SFRP1 in human osteoblasts accelerates the rate of osteoblast and osteocyte mortality. The lack of SFRP1 in mice leads to reduced osteoblast and osteocyte apoptosis (Bodine et al., 2005). Adult mice deficient in SFRP1 showed resistance to age‐related bone loss and improved fracture repair by promoting early bone union (Bodine et al., 2004; Gaur et al., 2009). In addition to osteoblast formation, SFRP1 can directly bind to RANKL and inhibit osteoclast formation (Hausler et al., 2004). Moreover, a small molecule inhibitor (diarylsulfone sulfonamide) that can bind and inhibit SFRP1 was shown to stimulate Wnt/β‐catenin signaling to increase bone formation (Bodine et al., 2009). All these confirmed the importance of SFRP1 for bone formation, and suggested that inhibition of SFRP1 may be a potential therapeutic target for increasing bone formation.

Several evidences strongly suggest that SFRP1 is a functional target of miR‐542‐3p and mediates its regulatory role in osteoblast formation. First, SFRP1 may be a possible target with 9 nt consistent matching site complementary to miR‐542‐3p in the 3′UTR of sFRP1. Second, luciferase reporter assay showed that overexpression of miR‐542‐3p inhibited the activity of pGL3‐sFRP1‐3′UTR‐WT, whereas pGL3‐sFRP1‐3′UTR‐Mut eliminated this inhibitory effect. Third, the level of SFRP1 protein was decreased after overexpression of miR‐542‐3p. Finally, during the process of MSCs differentiation, the SFRP1 protein showed a higher increase in OVX rats with a lower miR‐542‐3p expression.

Then we observed the in vitro effect of miR‐542‐3p on osteoblast differentiation using rat MSCs. MiR‐542‐3p enhanced the expression of osteoblast specific markers. And we further investigated the in vivo effects of miR‐542‐3p on ovariectomy‐induced osteoporosis in rats. Inhibition of miR‐542‐3p promoted SFRP1 protein expression, decreased bone mass and bone formation, aggravated bone loss in OVX rats. Whereas expression of miR‐542‐3p mutant did not inhibit SFRP1 protein expression, bone mass and bone formation in OVX rats. These data suggested that miR‐542‐3p influenced bone mass by regulating bone formation in vivo, primarily through its effect on SFRP1.

In conclusion, this study provides evidence that miR‐542‐3p in rat MSCs regulate osteoblast formation by targeting sFRP1. Furthermore, reduction of miR‐542‐3p expression in OVX rats results in attenuated osteoblast formation and contributes to osteoporosis. Together, our study has revealed that miR‐542‐3p plays an important role in the pathogenesis of postmenopausal osteoporosis and contributes to the development of a new therapeutic approach for osteoporosis.

## 4 MATERIALS AND METHODS

This study was approved by the Medical Ethical Committee of Kunming Medical University (Yunan, China). The written informed consents were obtained from all the participants enrolled in the study. And personal information on demographic factors was collected by structured questionnaire. All experiment methods were performed in accordance with the relevant guidelines and regulations.

### 4.1 Animal studies

All procedures were approved by the Animal Care and Use Committee of Kunming Medical University. Healthy specific‐pathogen‐free (SPF) female Sprague–Dawley (SD) rats were purchased from the Vital River. All rats were preserved under standard housing laboratory conditions. After 1 week of adaptation to the diet and the new environment, the rats were randomly divided into two groups: OVX group and sham‐operated group (Sham). All surgeries were performed by one person under anesthesia by ketamine 10% (100 mg/kg, Alfasan, Netherlands) and xylazine 2% (10 mg/kg, Alfasan, Netherlands). 45 or 90 days post‐surgery, rats were weighed again and anesthetized with ketamine and xylazine solution intraperitoneally and sacrificed by using thiopental (100 mg/kg) at the end of the experiment. The left femora and the L2‐5 vertebrae with the skin and muscle removed were kept in physiological saline and stored at −20°C for detection of BMD and for biomechanics procedures. For protein and RNA analysis, the right femora, L1 vertebrae and tibiae were frozen in liquid nitrogen, and stored at −80°C until use.

### 4.2 Reagents and antibodies

SFRP1 and β‐actin antibody were purchased from Abcam (Cambridge, UK). MiR‐542‐3p mimic/inhibitor/inhibitor mutant and non‐specific control were obtained from RiboBio (Gua ngzhou, China). Lipofectamine 2000 was purchased from Invitrogen.

### 4.3 Cell culture

HEK293T cells were obtained from American Type Culture Collection. Primary bone marrow‐derived mesenchymal stem cells (MSCs) were isolated from 4‐month old SD female rat. The cell lines were cultured with DMEM containing 10% FBS, supplemented with 100 μg/ml streptomycin and 100 U/ml penicillin, at 37 °C in a humidified atmosphere of 5% CO_2_.

### 4.4 RNA isolation and quantitative real‐time PCR

Total RNA was isolated with TRIzol reagent (Invitrogen Life Technologies, Carlsbad CA) according to the manufacturer's protocol. cDNA was synthesized with a Reverse Transcription Kit (TOYOBO, Tokyo, Japan) according to the manufacturer's instruction. Quantitative real‐time PCR (qRT‐PCR) was carried out using ABI 7500. The following primers were used: GAPDH, forward: 5′‐TGGCCTTCCGTGTTCCTAC‐3′, reverse: 5′‐GAGTTGCTGTTGAAGTCGCA‐3′; secreted frizzled related protein 1 (SFRP1), forward: 5′‐TACTGGCCCGAGATGCTCAA‐3′, reverse: 5′‐GAGGCTTCCGTGGTATTGGG‐3′; alkaline phosphatase (ALP), forward: 5′‐TCAGGGCAATGAGGTCACAT‐3′, reverse: 5′‐CCTCTGGTGGCATCTCGTTA‐3′ runt related transcription factor 2 (RUNX2), forward: 5′‐GACTGTGGTTACCGTCATGGC‐3′, reverse: 5′‐ACTTGGTTTTTCATAACAGCGGA‐3′ osteocalcin (OCN), forward: 5′‐CCCTGAGTCTGACAAAGCCT‐3′, reverse: 5′‐GCGGTCTTCAAGCCATACTG‐3′.

### 4.5 RNA‐seq library preparation and sequencing

Total RNA was extracted by the same method used for qRT–PCR. The RNA quality was checked by a 2,100 Bioanalyzer (Agilent Technologies, Santa Clara, CA) and only high quality samples were used to construct the sequencing library. Then RNA‐seq libraries were sequenced using the Illumina HiSeq 2,000 Sequencing System. Read counts mapped to each gene were calculated by HTseq (http://www-huber.embl.de) with the default model. Fragments per kilobase of exon model per million fragments mapped values were calculated using Cufflinks (version 2.1.1, http://cufflinks.cbcb.umd.edu). Differential expression was analysed with R (v2.14.0, http://www.R-project.org) and Bioconductor (release 2.10) with the R package DESeq (v1.6.0). miRNA targets were predicted by miRWalk2.0 (http://zmf.umm.uni-heidelberg.de/apps/zmf/mirwalk2/index.html).

### 4.6 Western blot

Cells were lysed in RIPA buffer plus protease inhibitors (Roche, Indianapolis, IN). Equal amount of proteins were loaded, and separated on 10% SDS–PAGE and then transferred to a nitrocellulose (NC) membrane (BD), blocked by incubation with 5% fat‐free milk in TBST buffer (150 mM NaCl, 50 mM Tris‐HCl, 0.5% Tween20, pH 7.6) at room temperature for 1 hr. The membranes were incubated with primary antibodies at 4 °C overnight, and then were incubated with horseradish peroxide‐conjugated secondary antibodies at room temperature for 1 hr. The blots were developed with ECL reagent (Thermo Fisher Scientific, Pierce, Rockford, IL).

### 4.7 Luciferase assays

The 3′UTR of *SFPR1* was amplified using the following primers: forward: 5′‐CTGGTTGATTCACTCAAGAGTTC‐3′, reverse: 5′‐TATCTGCTGGCAACAGGTCAGAAC‐3′. The PCR product was purified and then inserted into pGL3 within Xho I and Not I restriction sites. The QuikChange site‐directed mutagenesis kit (Stratagene, La Jolla, CA) was used to introduce mutations into the seed region of pGL3‐sFRP1‐3′UTR‐WT. HEK293T cells were co‐transfected with 0.5 µg of the reporter vector (pGL3‐sFRP1‐3′UTR‐WT or pGL3‐sFRP1‐3′UTR‐Mut) and 1 µg of miR‐542‐3p mimic or non‐specific control. Forty‐eight hours after transfection, cells were harvested and luciferase activity was detected using a dual‐luciferase reporter assay system (Promega, Madison, WI).

### 4.8 Bone mineral density measurement

Bone mineral density (BMD) of the femur at the end of the experiment by Lunar Prodigy Advance (GE Healthcare, Pittsburgh, PA) using appropriate software specifically for rats (Pastoureau, Chomel, & Bonnet, 1995). Results are given in g/cm^2^.

### 4.9 Bone histomorphometric analysis

For histological analyses, the femurs were fixed in 70% ethanol, embedded in methyl methacrylate, and serial sectioned into 5‐µm by a microtome. The parameters obtained for the bone formation were BFR/BS and Ob.S/BS. The parameters measured for bone resorption were Oc.S/BS and N.Oc/B.Pm.

After euthanasia, the femurs were resected and fixed in 10% formalin for 48 hours, decalcified in 10% ethylenediamine tetraacetic acid (EDTA) (pH 7.0) for 14 days and embedded in paraffin. Hematoxylin and eosin (HE) staining was performed according to the previous study (Huang, Yang, Hsieh, & Liu, 2002).

### 4.10 Statistical analysis

Data are presented as mean ± standard deviation (SD). The statistical analysis was performed using Student *t* test or one‐way ANOVA. All experiments were repeated at least three times, and representative experiments are shown. Differences were considered significant at *p* < 0.05.

## CONFLICT OF INTEREST

The authors declare no competing financial interests.

## Supporting information

Additional Supporting Information may be found online in the supporting information tab for this article.

Supporting Tables S1.
